# Oral versus intravenous antibiotics: Oral antibiotics are more cost-effective and may be safer than intravenous antibiotics for most infections in stable adults

**DOI:** 10.4102/phcfm.v17i1.5282

**Published:** 2025-12-13

**Authors:** Davie Wong, Thomas Perry

**Affiliations:** 1 Department of Anaesthesiology, Pharmacology and Therapeutics, Faculty of Medicine, University of British Columbia, Vancouver, Canada

**Keywords:** oral antibiotics, intravenous antibiotics, antibiotic overuse, efficacy, safety, therapeutics

## Abstract

Many clinicians perceive intravenous (IV) antibiotics as inherently more effective than their oral counterparts. However, randomised controlled trials (RCTs) have demonstrated that oral antibiotics are clinically equivalent to IV antibiotics for many severe bacterial infections. This includes pneumonia, skin and soft tissue infections, pyelonephritis, intra-abdominal infections, osteoarticular infections, bacteraemia and infective endocarditis. When clinically appropriate, oral treatment is more patient-friendly, cost-effective and environmentally friendly. But we still use the IV route much more than necessary. To address a historical practice that is often unwarranted, this *Therapeutics Letter* reviews evidence from RCTs and compares the advantages and disadvantages of oral and IV antibiotics. We suggest criteria to determine when oral therapy is appropriate.

## Vignette

*A 65-year-old woman bumped her left leg on a chair at home. A day later, she developed a rapid onset of pain and swelling. She attends an emergency department with a temperature of 38.5 °C, heart rate 82/min and blood pressure 124/76. Her lower leg is diffusely red, hot and tender to palpation. She weighs 60 kg and has normal kidney function. You diagnose non-purulent cellulitis and decide that she can be treated as an outpatient with a cephalosporin. Should you prescribe an oral or an intravenous antibiotic?*

## Introduction

The discovery of penicillin revolutionised the treatment of bacterial infections. People who would otherwise have died experienced miraculous cures. Because penicillin was valuable and poorly absorbed when taken orally, it was often given by injection. Convenient intravenous (IV) infusion equipment soon made it easy to deliver drugs intravenously.

Subsequent development of several poorly absorbed oral antibiotics, which proved less efficacious than penicillin, led medical experts to conclude that IV therapy was inherently superior to oral treatment.^1^ For decades, this dogma was unchallenged, and it underpinned many authoritative recommendations on infection management. But by the 1990s, randomised controlled trials (RCTs) began to provide high-quality evidence challenging the generalisation. By 2001, the British Thoracic Society recommended initial oral antibiotics for all but the most severe cases of pneumonia requiring hospitalisation.^2^

## Evidence for oral antibiotic efficacy

No RCT has proven IV administration superior to an equivalent oral antibiotic.^3^ On the other hand, in appropriately selected patients, evidence from individual RCTs and systematic reviews and meta-analyses supports the clinical equivalence of oral administration, whether as initial treatment or as transitional therapy after initial IV treatment. This includes people hospitalised for *Staphylococcus aureus* bacteraemia,^4,5^ Gram-negative bacteraemia,^6^ bone and joint infections,^7,8,9^ complicated urinary tract infections,^10^ intra-abdominal infections,^11^ skin and soft tissue infections,^11,12,13^ pneumonia,^14,15^ many people with febrile neutropenia^16^ and even for infective endocarditis.^17,18^ Surprisingly, but for reasons yet to be elucidated, oral therapy was associated in some RCTs with better outcomes in infective endocarditis,^19^ Gram-positive bacteraemia^20^ and moderate-to-severe cellulitis.^21^

In contrast, for most central nervous system infections, there is currently no RCT evidence for oral therapy. One exception, in patients able to swallow or tolerate a gastric tube, is the standard four-drug oral regimen for tuberculous meningitis (rifampicin, isoniazid, pyrazinamide, ethambutol).^22^ Intrinsic chemical properties of a drug, not the route of administration, determine its ability to penetrate the blood–brain barrier and other tissues.^23^ In RCTs, the oral and IV antibiotic comparators are often from different drug classes with different pharmacologic properties. When experiments randomise patients not only to the route of administration but also to different antibiotic classes, differences in outcomes can relate to either or both variables.^24,25^ To determine whether the mode of administration makes a difference, oral and IV antibiotics should be from the same drug class.

## Is initial intravenous treatment or a minimum intravenous duration evidence based?

In severe bacterial infection (sepsis or septic shock), when cardiovascular collapse may be imminent, urgent delivery of antibiotics through the bloodstream remains imperative. The IV route guarantees not only speed but also complete absorption, and some IV antibiotics offer a broader spectrum of activity than their oral counterparts.

But what about infections that pose no immediate threat to life or limb? Many clinicians were taught and still prefer initial IV antibiotics, with some minimum duration before a change to oral treatment. However, treatment durations are inherently arbitrary, and we lack controlled trial evidence to support this practice.^3,26^ Many antibiotics have excellent oral bioavailability,^27^ and evidence is mounting that initial oral treatment or a rapid switch from IV to oral is appropriate.^26^ Recent examples include:

- An Australian RCT (*N* = 47) in adults with moderate-to-severe cellulitis and clinical evidence of systemic infection deemed to require IV therapy found similar cure rates with initial oral or IV treatment.^21^
- A European RCT (*N* = 213) in uncomplicated *S. aureus* bacteraemia, in which after 5–7 days of IV antibiotics, a switch to oral therapy produced outcomes similar to ongoing IV treatment.^4^
- A Swiss RCT (*N* = 141) in hospitalised patients with serious urinary tract infections (including bacteraemia) showed that initial empiric oral ciprofloxacin was as effective as IV ciprofloxacin.^10^
- A Dutch nested cohort study matched by propensity scores for complications in emergency department patients (*N* = 173) with moderate-to-severe community-acquired pneumonia. Results of oral antibiotic treatment did not differ from IV treatment.^28^
- A Swiss RCT of diabetic foot osteomyelitis (*N* = 93) in which, after a median of 2 days of IV antibiotics, 3 weeks of oral antibiotics did not differ from 6 weeks.^29^

These examples challenge an established belief about infection management that remains very influential: that systemically ill patients should initially receive antibiotics intravenously – with transition to oral antibiotics only once they have improved. However, in febrile patients who are not critically ill, the acute phase of infection neither impairs absorption of oral antibiotics nor reduces total antibiotic exposure measured as area under the curve (AUC), nor alters the probability of clinical success.^30^ As for other drugs, vomiting sometimes necessitates parenteral administration.

It is now well recognised that the immune response greatly influences the clinical picture of patients during infection. This includes clinical signs (fever, tachycardia, chills and rigours) and laboratory markers (leucocytosis, elevated CRP [C-reactive protein]). But symptoms and signs correlate poorly with pathogen burden.^31,32^ Thus, the observed immune reaction can be misleading as to whether an infection is improving or not. For example, paucibacillary infections such as cellulitis can trigger a powerful inflammatory response with local redness, heat, pain and swelling, with or without fever.^33^

In contrast, life-threatening invasive infections such as cryptococcal disease in acquired immunodeficiency syndrome (AIDS) patients can lack symptoms and signs of inflammation.^34^ Is it logical to assume that the intensity of the immune response should dictate the route of drug delivery? It may seem inherently more likely that profound immunocompromise warrants IV over oral antibiotic therapy – but this has not been investigated in RCTs.

## Intravenous antibiotics are overused

In Canada, the United States (US), Australia, South Africa, China and elsewhere, outpatient IV antibiotics are overused for both children and adults.^35,36,37,38,39^ A retrospective review of outpatients treated with IV antibiotics at a US Veterans Health Administration hospital in 2012 (*N* = 148) identified 30% who could have been managed with oral therapy. Another 11% received unneeded antibiotics. Even in those who received an infectious disease consultation, IV therapy was potentially avoidable in 22% of cases.^36^

This suggests that even experts in infection overprescribe IV antibiotics. British Columbia is no exception. In our largest Health Authority, pharmacists audited 200 randomly selected charts of patients hospitalised during 2019–2020 who received 10 high bioavailability antibiotics or antifungals. They found that half the patients treated with IV antibiotics could have been treated orally.^40^ During 2024, an infectious disease specialist audited a convenience sample of 100 outpatient IV antibiotic prescriptions from the emergency department at a single hospital. He concluded that for 59% of the patients, IV treatment could have been avoided.^41^

## Comparative benefits and harms

In hospital, IV medications require pharmacy preparation and nursing administration, increasing clinical workload.^42^ Starting with oral antibiotics, or switching from IV to oral, may also allow earlier hospital discharge.^4,43^ Oral treatment improves patient convenience, as freedom from an IV line increases mobility. It avoids frequent trips for outpatient infusions and unnecessary medical services and costs. When used appropriately, oral medications are far more cost-effective.^44^ Using oral rather than IV drugs also reduces their carbon footprint.^45,46^

The instantaneous and complete bioavailability of IV antibiotics is believed to be critical for patients with sepsis or shock because of concern that the absorption or distribution of antibiotics can be altered by systemic inflammation.^47^ But outside of critical illness, such pharmacokinetic advantages do not translate to better efficacy.

On the other hand, IV administration can cause harm. These include infusion rate errors, the use of an incorrect diluent and the volume of diluent infused, something particularly important in small children.^48^ Peripherally inserted central lines for prolonged antibiotic treatment increase adverse events such as vein thrombosis, superficial thrombophlebitis, infection, drug extravasation and contact dermatitis from adhesives.^49^ Intravenous treatment may cause fewer adverse events in the upper intestine. But with the exception of narrow-spectrum antibiotics like penicillin or aminoglycosides, it does not reduce antibiotic-associated diarrhoea nor spare the colonic microbiome.^7,17^ The intestine is not a sanctuary from antibiotics circulating in the bloodstream.

In Canadian public outpatient infusion clinics, unnecessary administration of IV antibiotics may displace or delay treatment of other patients for whom drug infusions are essential: for example, IV iron or biological drugs that cannot be taken by mouth.

## Which route do patients prefer?

If clinicians recommend IV antibiotics as more effective than oral, most patients will be reluctant to disagree. But when advised of equal efficacy, most will choose the oral route.^50^ An informed preference for oral treatment has also been shown for the treatment of rheumatoid arthritis and cancers.^51,52^ Patients prefer oral medications for the obvious advantages of convenience, earlier discharge from hospital and avoiding pain related to inserting and maintaining IV access.

## When is oral therapy more appropriate than intravenous therapy?

A decision to prescribe oral or IV therapy is often arbitrary and based on a clinician’s personal practice habits or local medical standards that are not evidence based.^53^ When oral treatment is deemed safe and effective, *Choosing Wisely Canada* recommends against IV treatment.^54^ Published criteria for transition from IV to oral administration can assist in determining whether it is appropriate to start with oral therapy:^55,56^

1. There is a safe and effective oral option.
2. Patient can swallow and absorb oral medication.
3. Patient is clinically stable: resolution of shock, no worsening symptoms and signs of infection (other than those expected from inflammation in conditions like cellulitis), clearance of blood cultures if relevant, inflammatory markers not increasing.
4. No source control problem requires intervention: abscess, foreign body or implant, infected heart valve.
5. No psychosocial reason to prefer IV therapy: patient cannot afford oral treatment or declines to take antibiotics by mouth.

An external reviewer of this *Therapeutics Letter* points out that – in programmes tailored to their specific needs – successful oral antibiotic therapy has been demonstrated even for unhoused people with multiple challenges.

## Will we know more in future?

Results of Swiss RCTs in diabetic foot infections that randomised over 400 participants and used mostly oral antibiotics are expected during 2025.^57^ And a major international RCT is assessing 90-day total mortality after early oral switch from IV antibiotics in uncomplicated and complicated *S. aureus* bacteraemia.^58^ It aims to recruit at least 1000 participants. This may become a model for collaborative trials that improve our understanding of optimum antibiotic therapy for dangerous infections.

## Recommendations

- For most stable patients, oral antibiotics should be the standard of care.
- Reserve IV therapy for critically ill patients and situations where oral administration is not possible or is not supported by evidence.
- In patients initially prescribed IV treatment, convert to oral therapy as soon as clinically appropriate.

## Vignette resolution

*Judging that your patient can safely and reliably take an oral antibiotic, you prescribe cephalexin 500 mg PO* [orally] *QID* [6 hourly] *for 5 days. Over the next 24 h, inflammation of her left leg worsens, and she returns to the emergency department for reassessment. Now she requests an IV antibiotic because she thinks the oral treatment is not working. But she is afebrile, and her other vital signs are normal. You advise continuing the oral cephalexin, pointing out that symptoms or signs of infection can worsen before they get better, as dying bacteria release their toxins. Elevating the leg and taking analgesics will reduce her inflammatory symptoms. Recognising that you reassessed her carefully, she accepts your recommendation. Her soft tissue infection resolves gradually – vindicating your evidence-based approach*.

## Summary and conclusions

Medical training and guidelines often still encourage unnecessary use of IV antibiotics, an obstacle to more efficient and patient-friendly therapy. The route of drug delivery is often overemphasised, distracting us from more important aspects of infection management. Strategies to promote oral therapy include education of clinicians and patients, prescription of antibiotics with infrequent dosing schedules to improve adherence, electronic medical record prompts for early IV to oral switch and easy access to local guidelines.^59^

Removing financial incentives that encourage clinicians and hospitals to prescribe outpatient IV antibiotics could help, especially when oral treatment is publicly funded. When circumstances and evidence suggest that oral therapy could be preferable, this *Therapeutics Letter* may encourage more clinicians to consider moderating our historical preference for IV treatment of most serious infections:

- Oral antibiotics are as effective as IV antibiotics for most bacterial infections.
- For stable patients, oral antibiotics should be first-line therapy. Reserve IV treatment for people who cannot take pills or for infections for which effective oral therapy does not exist.
- Oral treatment improves patient experience, consumes fewer health system resources and generates a lower carbon footprint.
